# Aerobic Vitamin B_12_ Biosynthesis Is Essential for *Pseudomonas aeruginosa* Class II Ribonucleotide Reductase Activity During Planktonic and Biofilm Growth

**DOI:** 10.3389/fmicb.2018.00986

**Published:** 2018-05-15

**Authors:** Anna Crespo, Núria Blanco-Cabra, Eduard Torrents

**Affiliations:** Bacterial Infections and Antimicrobial Therapies, Institute for Bioengineering of Catalonia, Barcelona Institute of Science and Technology, Barcelona, Spain

**Keywords:** Vitamin B_12_, adenosylcobalamin, ribonucleotide reductases, *Pseudomonas aeruginosa*, NrdJ, bacterial growth, biofilm, anaerobiosis

## Abstract

*Pseudomonas aeruginosa* is a major pathogenic bacterium in chronic infections and is a model organism for studying biofilms. *P. aeruginosa* is considered an aerobic bacterium, but in the presence of nitrate, it also grows in anaerobic conditions. Oxygen diffusion through the biofilm generates metabolic and genetic diversity in *P. aeruginosa* growth, such as in ribonucleotide reductase activity. These essential enzymes are necessary for DNA synthesis and repair. Oxygen availability determines the activity of the three-ribonucleotide reductase (RNR) classes. Class II and III RNRs are active in the absence of oxygen; however, class II RNRs, which are important in *P. aeruginosa* biofilm growth, require a vitamin B_12_ cofactor for their enzymatic activity. In this work, we elucidated the conditions in which class II RNRs are active due to vitamin B_12_ concentration constraints (biosynthesis or environmental availability). We demonstrated that increased vitamin B_12_ levels during aerobic, stationary and biofilm growth activate class II RNR activity. We also established that the *cobN* gene is essentially responsible for B_12_ biosynthesis under planktonic and biofilm growth. Our results unravel the mechanisms of dNTP synthesis by *P. aeruginosa* during biofilm growth, which appear to depend on the bacterial strain (laboratory-type or clinical isolate).

## Introduction

*Pseudomonas aeruginosa* is an opportunistic pathogen that causes severe chronic infections in immunocompromised patients and other risk groups, such as cystic fibrosis (CF) or chronic obstructive pulmonary disease (COPD) patients. The key to *P. aeruginosa* survival in environments that range from soil to various living host organisms is its metabolic versatility. It subsists on various carbon sources for energy, uses nitrogen as a terminal electron acceptor under anaerobic conditions, requires minimal nutrients, and grows at temperatures up to 42°C. *P. aeruginosa* uses anaerobic metabolism to reduce nitrogen (N_2_) via the denitrification process (Schobert and Jahn, 2010; Arat et al., 2015), as an essential metabolic condition during chronic infection and biofilm growth (Yoon et al., 2006; Hassett et al., 2009; Crespo et al., 2016).

During *P. aeruginosa* infections, bacteria must multiply inside the infected organisms (plant, animal, insect, etc.), requiring active DNA synthesis for bacterial cell division. Ribonucleotide reductase (RNR) enzymes provide all living organisms with deoxyribonucleotide triphosphates (dNTP) supplying the monomers for DNA synthesis. Three different RNR classes exist (class I, subdivided into Ia, Ib, and Ic; class II and class III) that differ in their overall protein structure and cofactor requirements, but all possess allosteric regulation and use organic radicals to initiate catalysis through free radical chemistry (Jordan and Reichard, 1998; Cotruvo Jr and Stubbe, 2011; Hofer et al., 2012; Torrents, 2014). *P. aeruginosa* is one of the few organisms that encode three different RNR classes; the oxygen-dependent class Ia (encoded by the *nrdAB* genes), the oxygen-independent class II (encoded by the *nrdJab* genes) and the oxygen-sensitive class III (encoded by the *nrdDG* genes). Specifically, class II RNR activity depends on an external cofactor, adenosylcobalamin (AdoCob) or vitamin B_12_, to generate its radical independently of oxygen to reduce the different ribonucleotides to their corresponding deoxyribonucleotides.

Vitamin B_12_ is one of the most structurally complex cofactors synthesized by bacteria (Warren et al., 2002); however, not all microorganisms encode for the ~25 genes needed for the complete biosynthetic pathway. In nature, two vitamin B_12_ biosynthesis pathways exist: the aerobic, or late cobalt insertion pathway and the anaerobic, or early cobalt insertion pathway (Warren et al., 2002). One of the genes involved in the aerobic pathway that participates in cobalt insertion is the *cobN* gene described extensively in *Pseudomonas denitrificans* (Warren et al., 2002). The most studied anaerobic biosynthetic pathway involved in early cobalt insertion was described in *Salmonella typhymurium* (Roth et al., 1993).

*Pseudomonas* synthesizes vitamin B_12_ for different metabolic reactions, such as methionine synthesis, cobalamin biosynthesis, and RNR enzymes. One essential reaction is ribonucleotide synthesis by RNR. *P. aeruginosa* PAO1 has been demonstrated to grow in a filament cell morphology due to cellular stress by RNR activity depletion, such as the low expression levels of class III RNR under anaerobic conditions (Lee et al., 2012; Crespo et al., 2017) or the high nitric oxide levels in the denitrification process, which interacts with a cobalamin precursor of the vitamin B_12_ pathway (Broderick et al., 2005; Yoon et al., 2011; Sullivan et al., 2013). Therefore, this cell filamentation results from DNA replication impairment that affects *P. aeruginosa* PAO1 cell division, thus affecting infection (Sjöberg and Torrents, 2011; Crespo et al., 2017), anaerobic growth (Torrents et al., 2005; Torrents, 2014) and biofilm growth (Crespo et al., 2016). Class II and III RNR enzymes reduce ribonucleotides under these conditions. Thus, their activity is essential for cell division (Crespo et al., 2016, 2017).

Class II RNR (NrdJab) activity is oxygen independent, but it strictly depends on vitamin B_12_ availability. To date, the link between internal vitamin B_12_ biosynthesis or availability from the environment and the real class II RNR activity is unknown. Therefore, in this work, we analyzed *P. aeruginosa* vitamin B_12_ biosynthesis during aerobic growth, anaerobic growth and biofilm formation. We also determined the relationship between vitamin B_12_ biosynthesis and class II RNR activity under different growing conditions.

## Materials and methods

### Bacterial strains and growth conditions

*Pseudomonas aeruginosa* and *Escherichia coli* strains, listed in Table 1, were grown in Luria-Bertani broth (LB) or minimum medium (MM) (Kjaergaard et al., 2000) at 37°C. MM containing 1% KNO_3_ (MMN) was used for anaerobic liquid growth in screw-cap tubes (Hungate tubes) (Garriga et al., 1996; Crespo et al., 2016) or in anaerobic plates using the GENbag system (bioMérieux) according to the manufacturer's instructions.

**Table 1:** Strains and plasmids used in this study.

**Strain or plasmid**	**Description**	**Source**
**PLASMIDS**		
pJET1.2/blunt	*Blunt-end vector, AmpR*	*Thermo Scientific*
pUCGmlox	Ap^R^, Gm^R^; source of Gm^R^ cassette	Hoang et al., 1998
pEX18Tc	*sac*B based counter-selection vector, Tc^R^	Hoang et al., 1998
pETS160	pBBR1 derivative carrying *nrdDG* operon, Gm^R^	Sjöberg and Torrents, 2011
pETS134	pETS130 derivative carrying *nrdA* promoter, Gm^R^	Sjöberg and Torrents, 2011
pETS136	pETS130 derivative carrying *nrdD* promoter, Gm^R^	Sjöberg and Torrents, 2011
pETS180	pETS130 derivative carrying *nrdJ* promoter, Gm^R^	Crespo et al., 2015
**STRAINS**		
***E. coli***
DH5α	*recA1 endA1 hsdR17 supE44 thi-1 relA1 Δ(lacZYA-argF)U169 deoR Φ80dlacZM15*	Lab stock
S17.1 λ*pir*	*recA thi pro hsdR- M+RP4::2-Tc::Mu::Km Tn7 Tpr Smr Xpir*	de Lorenzo et al., 1993
***P. aeruginosa***
PAO1	Wild-type (ATCC 15692 / CECT 4122)- Spanish Type Culture Collection	Lab strain
PA14	Wild-type *P. aeruginosa* PA14	Lab strain
PAET1	*CF strain isolated from chronic patient*	Lab strain, (Crespo et al., 2017)
ETS102 (Δ***nrdJ***)	*P. aeruginosa* PAO1 *nrdJ*::ΩTc; Tc^R^	Sjöberg and Torrents, 2011
ETS103 (Δ***nrdD***)	*P. aeruginosa* PAO1 *nrdD*::ΩTc; Tc^R^	Sjöberg and Torrents, 2011
ETS125 (Δ***nrdJ**Δ**nrdD***)	*P. aeruginosa* PAO1 *nrdD*::ΩTc; Tc^R^, *nrdJ*::ΩGm; Gm^R^	Crespo et al., 2016
ETS126 (Δ***cobN***)	*P. aeruginosa* PAO1 *cobN*::ΩGm, Gm^R^	This work

The medium was supplemented, when necessary, with the following antibiotics: 100 μg/ml or 50 μg/mL gentamicin, 300 μg/ml carbenicillin and 40 μg/ml tetracycline for *P. aeruginosa*, and 10 μg/ml gentamicin and 50 μg/ml ampicillin for *E. coli*.

### Construction of *cobN* deletion mutant strain

*Pseudomonas aeruginosa* PAO1 with a mutation in the *cobN* gene (ETS126; Δ*cobN*) was constructed by inserting the gentamicin-resistance gene (*aacC1*) into the *cobN* gene by homologous recombination using the pEX18Tc vector, as previously described (Quenee et al., 2005; Sjöberg and Torrents, 2011). Briefly, two 400-bp areas surrounding the *P. aeruginosa* PAO1 *cobN* gene were amplified by PCR using the High-Fidelity PCR Enzyme Mix (Thermo Scientific) with the primer pairs, CobN1HIII-up/CobN2BI-low and CobN3BI-up/CobN4SI-low, listed in Table 2. The two amplicons were cloned separately into the pJET1.2 vector (Thermo Scientific). A plasmid containing both fragments was generated by *BamHI/SacI* digestion. The gentamicin resistance gene *aacC1* was obtained using *Bam*HI digestion of pUCGmlox, and the corresponding cassette was ligated to the two fragments. The construct was cloned into the *sac*B gene-based counter-selection pEX18Tc vector and transferred into the S17.1λ*pir* strain for *P. aeruginosa* PAO1 conjugation as previously described (Crespo et al., 2016). Transconjugants were selected by plating them with tetracycline, gentamicin and sucrose (5%), used for *sacB*-mediated plasmid counter selection. *aacC1* insertion was screened and verified by PCR with the primer pair CobN1HIII-up/CobN5-low and later confirmed by DNA sequencing.

**Table 2:** Oligonucleotides and probes used in this study.

**Name**	**Sequence (5′ → 3′)**	**Application**
pJET-rev	AAGAACATCGATTTTCCATGGCAG	Check-Cloning
pJET-up	CGACTCACTATAGGGAGAGCGGC	Check-Cloning
CobN1HIII-up	AAGCTTATGCACCTGTTGCGCACCC	Cloning
CobN2BI-low	GGATCCCAGAAGCGCTCGGCCTGCT	Cloning
CobN3BI-up	GGATCCTGAATCCGAAGTGGATCGC	Cloning
CobN4SI-low	GAGCTCCTATTCCTCTTCCGACGTCCA	Cloning
CobN5-low	CAGGCCAGGCCCTTGAAAC	Cloning
gapTaqM-low	GAGGTTCTGGTCGTTGGT	qRT-PCR
nrdATaqM2-low	TGTTCATGTCGTGGGTACG	qRT-PCR
nrdJTaqM2-low	GTAAACACCCGCACCACTTC	qRT-PCR
nrdDTaqM2-low	CCGAGTTGAGGAAGTTCTGG	qRT-PCR
Univ-Res-Gen-lw	AAGAATTCACGCGTCGCTCATGAGACAATA	Cloning
Univ-Res-Gen-up	AAGAATTCACGCGTATATATGAGTAAACTT	Cloning
nrdA-FAM	CTGGCACCTGGACATC	qRT-PCR probe
nrdJ-FAM	TCGGCTCGGTCAACCT	qRT-PCR probe
nrdD-FAM	CCCGACCTACAACATC	qRT-PCR probe
gap-FAM	CCTGCACCACCAACTG	qRT-PCR probe

### Quantitative real-time PCR (qRT-PCR)

Transcripts of RNR genes (*nrdA, nrdJ* and *nrdD*) were quantified using quantitative real-time PCR (qRT-PCR). *P. aeruginosa* was grown in planktonic conditions at the mid-exponential growth phase in which samples were treated with RNAprotect Bacterial Reagent (Qiagen). The RNeasy Mini Kit (Qiagen) was used to isolate and purify total RNA, and extra DNA was removed using DNase I (Turbo DNA-free, Applied Biosystems) per the manufacturer's instructions. DNA contaminations were verified by PCR. cDNA was synthetized using 0.5 μg of RNA with SuperScript III Reverse Transcriptase (Thermo Scientific). The primers used are listed in Table 2 (Crespo et al., 2016). RNA was quantified using a NanoDrop 1000 spectrophotometer (Thermo Scientific). The *gapA* gene was used to normalize the transcript gene levels.

### Green fluorescent protein gene reporter assay

GFP fluorescence expressed in plasmids, pETS134 (P*nrdA*), pETS180 (P*nrdJ*) and pETS136 (P*nrdD*), was measured to determine each RNR gene's promoter activity. *P. aeruginosa* containing the *nrd* promoter fusion was grown to exponential phase, and three independent 1-ml samples were analyzed. Cells were fixed with 1 ml of freshly prepared PBS 1x solution containing 2% formaldehyde (Sigma) and stored in the dark at 4°C. GFP fluorescence was measured in a 96-well plate (Costar® 96-Well Black Polystyrene Plate, Corning) on an Infinite 200 Pro Fluorescence Microplate Reader (Tecan), as previously described (Crespo et al., 2015). Three measurements were performed per independent sample.

### Continuous-flow biofilm formation

Continuous-flow cell biofilms were grown in MM + 0.2% glucose and performed as previously described (Baelo et al., 2015; Crespo et al., 2016). These *in vitro* formed biofilms are a more natural, mature biofilm with clear oxygen concentration stratification (Stewart and Franklin, 2008). Briefly, biofilms were grown in a three-channel flow cell with a constant flow rate of 42 μl per minute for each channel using an Ismatec ISM 943 pump (Ismatec). After 5 days of growth, biofilms were stained with the LIVE/DEAD BacLight Bacterial Viability Kit (Thermo Scientific) and visualized using a Zeiss LSM 800 confocal laser scanning microscope (CSLM). Images were generated using ImageJ Fiji software, and COMSTAT 2 software was used to quantify biomass and biofilm thickness (Heydorn et al., 2000).

### Vitamin B_12_ quantification by HPLC-MS

*Pseudomonas aeruginosa* PAO1, PAET1, and PA14 strains were grown in MM or MMN medium for 20 h in aerobic or anaerobic conditions or for 5 days in a continuous-flow cell biofilm growth system. Cells were lysed using lysozyme (50 mg/ml) (Sigma) and sonicated five times on ice using a 6-mm sonication probe at 32% power for 20 s (Digital Sonifier, Branson). After centrifugation (4,000 × g at 4°C), the supernatants were filtrated with a 10-kDa Centricon column (Millipore). Samples were manipulated in the dark to avoid vitamin B_12_ degradation. Finally, 1% ammonium formate was added to each sample before HPLC-MS quantification. Samples of 10 μl were injected into the Luna 5-μm C18 100 Å (150 × 2 mm) column for HPLC-MS [4000 QTRAP (AB SCIEX) in an Aligent 1,200 Series] at the Separation Techniques platform of the Scientific Center Services of the Scientific Park of Barcelona (PCB). A calibration curve was constructed for vitamin B_12_ (Sigma) measured in the range of 0.1–100 ng/ml. Values were normalized, and the protein concentration was measured using a Bradford assay (Bio-Rad).

### Fluorescence microscopic imaging and analysis

*Pseudomonas aeruginosa* strain cultures were stained using the LIVE/DEAD BacLight viability kit (Thermo Scientific) for 15 min at room temperature in the dark. Fluorescent bacteria were visualized with a Nikon ECLIPSE Ti-S/L 100 inverted fluorescence microscope (Nikon) coupled with a Nikon DS-Qi2 camera. Live cells were visualized in green (SYTO 9 dye), and dead cells were visualized in red (propidium iodide dye). ImageJ software was used for image analysis.

## Results and discussion

### Vitamin B_12_ availability is essential for class II RNR activity in *P. aeruginosa* growth

Vitamin B_12_, or adenosylcobalamin (AdoCob), acts as a radical generator for class II RNR enzyme activity, but the link between vitamin B_12_ biosynthesis and class II RNR (NrdJ) activity in *P. aeruginosa* is poorly understood, and further investigations are required, especially during bacterial biofilm growth.

We first analyzed the essentiality and role of the class II RNR enzyme under aerobic and anaerobic conditions depending on vitamin B_12_ availability. We used diverse *P. aeruginosa* PAO1 strains deficient for different RNR classes (ETS102, Δ*nrdJ*; ETS103, Δ*nrdD*, and ETS125, Δ*nrdJ*Δ*nrdD*) (see Table 1). We also used a specific mutant strain for the vitamin B_12_ biosynthesis pathway involved in cobalt insertion under aerobic conditions (ETS126, Δ*cobN*). As the *nrdA* mutation is unviable (Sjöberg and Torrents, 2011), we added 30 mM hydroxyurea (HU) to mimic an *nrdA* mutant strain. Hydroxyurea interferes with *P. aeruginosa* PAO1 growth, arresting DNA replication by inhibiting NrdA activity (Gale et al., 1964; Sjöberg and Torrents, 2011; Lee et al., 2012; Julian et al., 2015).

**Figure 1:**
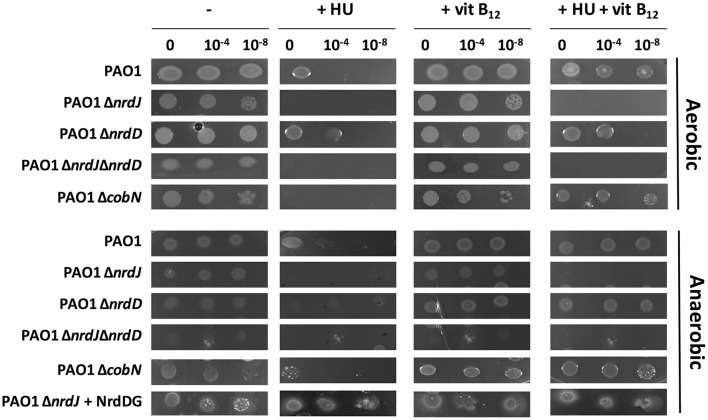
Effect of hydroxyurea and vitamin B_12_ on *P. aeruginosa* PAO1 wild-type, Δ*nrdJ*, Δ*nrdD*, Δ*nrdJ*Δ*nrdD*, and Δ*cobN* strain growth. Five-microliter drops were plated from a 0, 10^−4^, and 10^−8^ dilution into a solid medium containing 30 mM hydroxyurea (HU) and 1 μg/ml vitamin B_12_ (vit B_12_) for 48 h. Pictures represent three independent experiments.

Aerobically, class Ia RNR inhibition by HU decreased *P. aeruginosa* PAO1 wild-type growth in minimal medium, as previously described (Jordan et al., 1999; Torrents et al., 2005), but after 48 h of aerobic incubation, some growth was observed (see the undiluted sample 0 with HU in *P. aeruginosa* wild-type and Δ*nrdD*) (Figure 1). However, any *P. aeruginosa* with either a class II RNR or a vitamin B_12_ biosynthesis gene mutation (ETS102Δ*nrdJ*, ETS125Δ*nrdJ*Δ*nrdD*, and ETS126Δ*cobN*) treated with HU showed no growth after 48 h (Figure 1) or even after 72 h (data not shown). This result indicates that after 48 h of HU treatment, class II RNR remains active and allows *Pseudomonas* growth. Adding vitamin B_12_ into the minimal medium containing 30 mM HU, re-establishes the optimal aerobic growth in the strains encoding an active class II RNR (NrdJ) enzyme (*P. aeruginosa* PAO1 wild-type; ETS103, Δ*nrdD* and ETS126, Δ*cobN* strains). Therefore, vitamin B_12_ availability (from biosynthesis or the environment) supports class II RNR activity and rescues class Ia RNR deficiency by HU inhibition under aerobic conditions. Thus, in this work, we demonstrated that a *cobN* gene mutation disrupted vitamin B_12_ biosynthesis and completely abolished class II RNR activity, inhibiting aerobic bacterial growth (ETS126, Δ*cobN*).

However, under anaerobic conditions, class II and III RNR mutants (Δ*nrdJ*, Δ*nrdD*, and Δ*nrdJ*Δ*nrdD*) grew slightly less than the *P. aeruginosa* PAO1 wild-type and Δ*cobN* deficient strains (Figure 1). Δ*cobN* mutant strain growth was unaffected anaerobically (undiluted sample, 0). This result suggests that the *cobN* gene was uninvolved in *P. aeruginosa* vitamin B_12_ biosynthesis under anaerobic conditions. Another *Pseudomonas* strain, *P. denitrificans*, was shown to only synthesize vitamin B_12_ aerobically (Roth et al., 1996). Thus, *P. aeruginosa* PAO1 cannot sustain proper growth anaerobically unless RNR activity is increased by externally adding vitamin B_12_ to the medium (1 μg/ml) (this work) or by increasing class III RNR gene copy numbers by complementing extra external NrdDG copies [ETS103 (Δ*nrdD*)+pETS60 (+NrdDG)] as previously described (Crespo et al., 2017). Nevertheless, the vitamin B_12_ anaerobic internalization pathway remains unknown, and more experiments are required.

**Figure 2:**
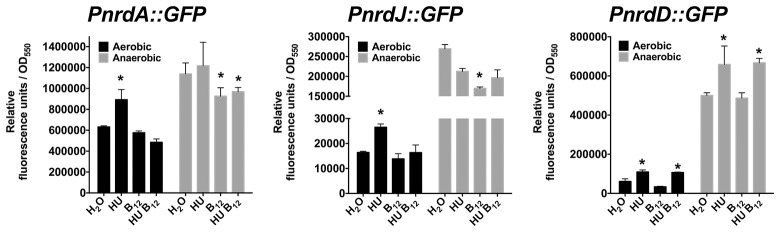
Expression analysis of the different *P. aeruginosa* PAO1 RNR classes under HU and vitamin B_12_ treatment under aerobic and anaerobic conditions. Cultures were treated in the presence of HU (30 mM) and vitamin B_12_ (1 μg/ml) for 20 min prior to measure the relative fluorescence units of *PnrdA* (pETS134)*, PnrdJ* (pETS180*)* and *PnrdD* (pETS136). The results are the mean of three independent experiments ± standard deviation. Asterisks over bars (^*^) indicate statistical differences compared to those without treatment (H_2_O) (*p* < 0.05 in pairwise *t*-test calculated with GraphPad 6.0).

Therefore, we demonstrated that class II RNR (NrdJ) is active in both aerobic and anaerobic conditions if vitamin B_12_ is available in the medium. However, class Ia and III RNR enzymes preferentially supply the dNTPs required for aerobic (Sjöberg and Torrents, 2011) and anaerobic (Crespo et al., 2017) bacterial DNA replication, respectively. Lack of class Ia and III RNR activity in planktonic culture, due to class Ia RNR activity inhibition by HU or by low *nrdD* expression levels, causes cell filamentation growth in *P. aeruginosa* PAO1 (Sjöberg and Torrents, 2011; Crespo et al., 2017), thus increasing its *nrd* expression (Figure 2). Adding vitamin B_12_ returns its cellular morphology to rod-shaped by restoring the DNA replication impairment by activating class II RNR (Crespo et al., 2017) and slightly decreasing expression of the three *nrd* genes (Figure 2), independently of B_12_-riboswitch regulation (Vitreschak et al., 2003). Other vitamin B_12_-dependent enzymes (methionine, cobalamin biosynthesis and some ribonucleotide reductases from other microorganisms) are usually regulated by a B_12_-riboswitch on their promoter regions (Vitreschak et al., 2003; Borovok et al., 2006).

**Figure 3:**
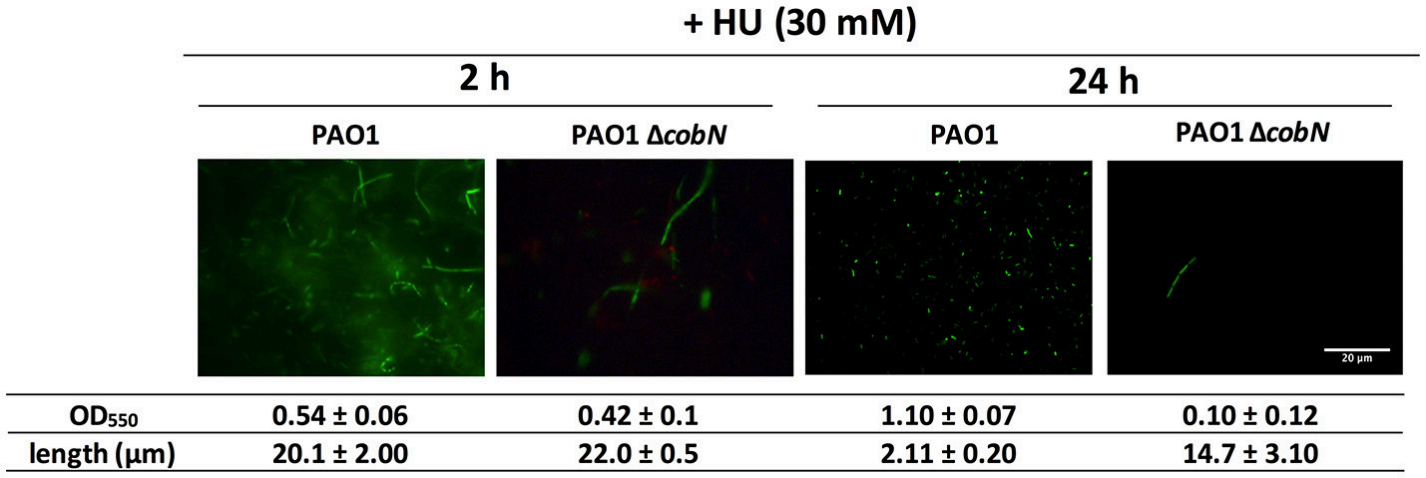
Cell morphology after hydroxyurea treatment. Fluorescence microscopy images from aerobic *P. aeruginosa* PAO1 wild-type and Δ*cobN* growth visualized after 2 and 24 h of HU treatment. Cells were stained with the LIVE/DEAD BacLight Bacterial Viability Kit, and the bacterial length was measured using ImageJ software. The images represent at least three different experiments. Scale bars, 20 μm.

In addition, *P. aeruginosa* PAO1 planktonic cells treated with HU for 2 h in minimal medium under aerobic conditions cause filamentous morphology (Figure 3); however, at 24 h post-HU treatment (late stationary phase), *P. aeruginosa* PAO1 cells return to their rod-shaped morphology without adding external vitamin B_12_ (Figure 3), indicating that DNA synthesis was restored only by class II RNR activity. Nevertheless, disrupting vitamin B_12_ biosynthesis using the *P. aeruginosa* PAO1 Δ*cobN* mutant strain causes filamentous cells even after 24 h of HU treatment. These results highlight an active vitamin B_12_ biosynthesis in *P. aeruginosa* PAO1 that specifically requires the *cobN* gene under aerobic stationary growing conditions for class II RNR activity and thus for DNA biosynthesis. However, vitamin B_12_ levels are insufficient during the initial hours of *P. aeruginosa* PAO1 growth (2 h) and likely reach optimal physiological levels of vitamin B_12_ after 24 h. Vitamin B_12_ biosynthesis pathway regulation requires further investigation.

**Figure 4:**
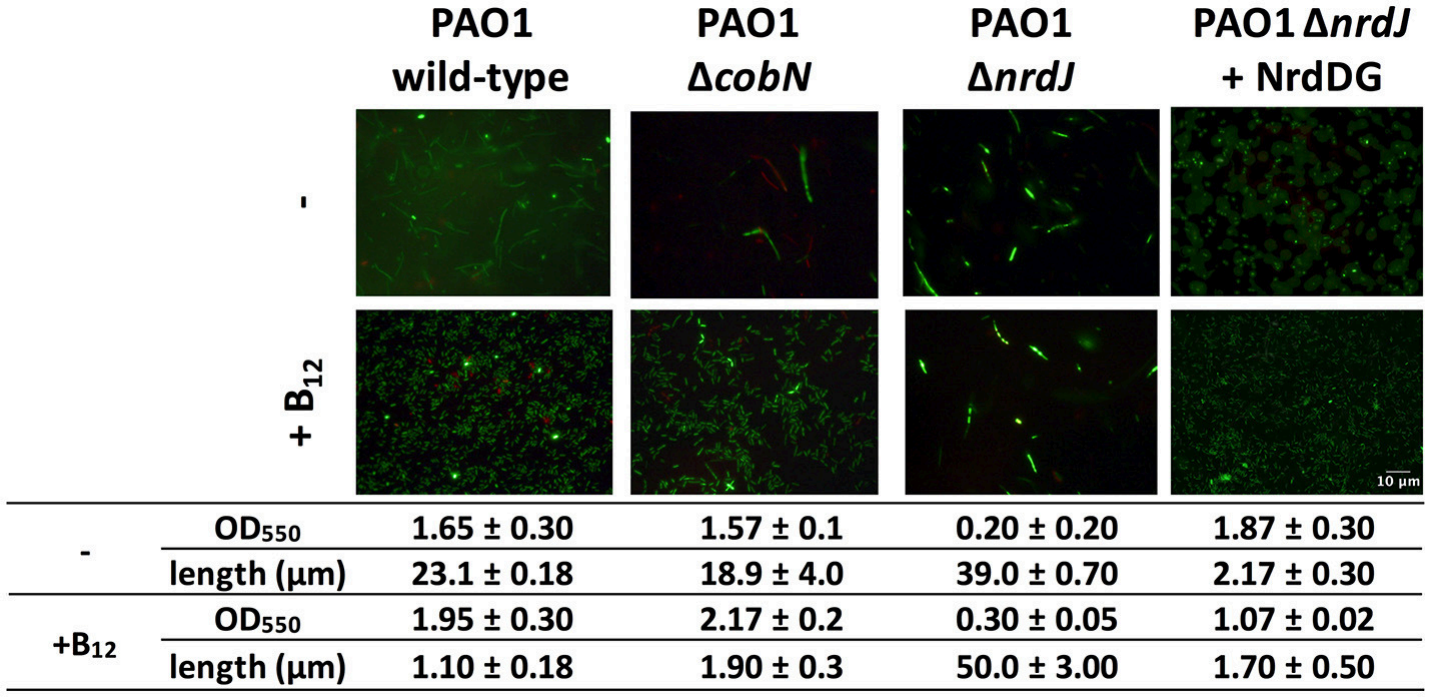
CobN is not involved in *P. aeruginosa* PAO1 anaerobic growth. Fluorescence microscopy pictures of *P. aeruginosa* PAO1 wild-type, Δ*cobN*, Δ*nrdJ, and* Δ*nrdJ* + NrdDG cells stained with the LIVE/DEAD BacLight Bacterial Viability Kit at 16 h of anaerobic growth in MM ± vitamin B_12_. The length was measured using ImageJ software. Scale bars, 10 μm.

*Pseudomonas aeruginosa* cell morphology under anaerobic conditions was filamentous due to the low class III RNR activity (Crespo et al., 2017). We also observed that the *P. aeruginosa* PAO1 Δ*cobN* mutant strain cell morphology was similar to the *P. aeruginosa* PAO1 strain, suggesting no vitamin B_12_ biosynthesis during anaerobic growth, even after 16 h (Figure 4). Hence, in anaerobic conditions, the *P. aeruginosa* PAO1 and Δ*cobN* strains growth needed external vitamin B_12_ supplementation for optimal class II RNR activity. This was demonstrated previously in the anaerobic *P. aeruginosa* PAO1 growth that was restored with an extra copy of *nrdDG* genes or by adding vitamin B_12_, enhancing RNR activity (Crespo et al., 2017).

### Biofilm formation depends on vitamin B_12_ synthesis

**Figure 5:**
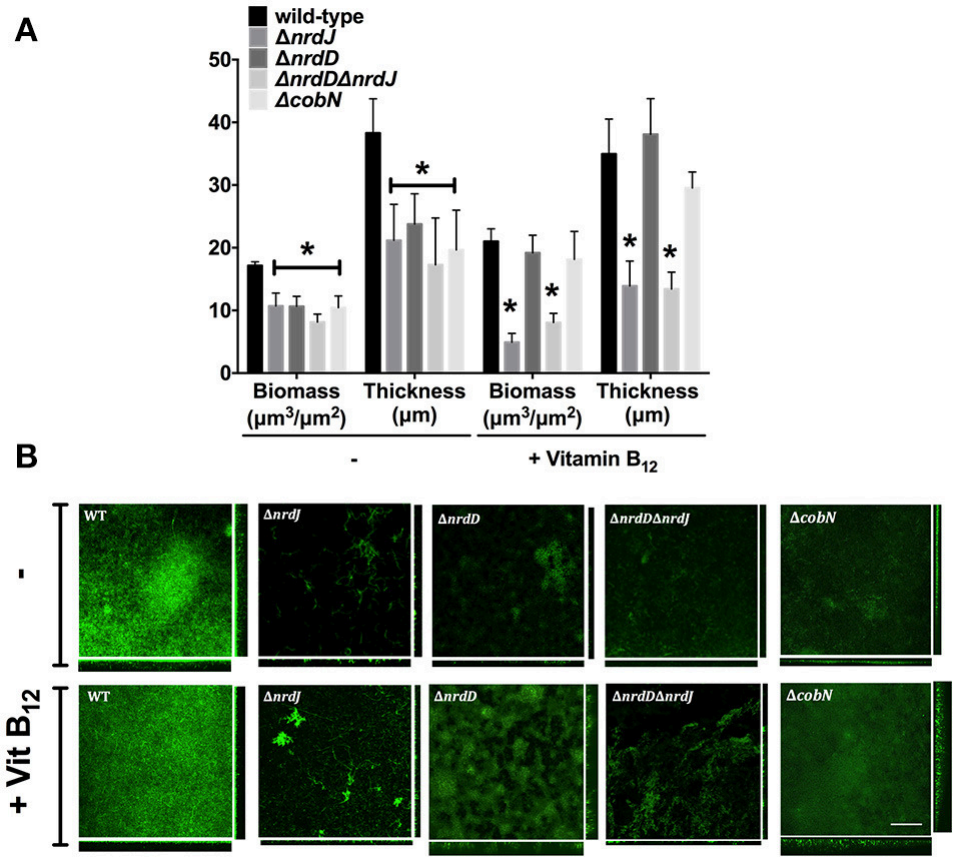
Vitamin B_12_ enables *P. aeruginosa* biofilm formation though class II RNR activity. **(A)** The continuous-flow cell biofilm of *P. aeruginosa* PAO1 wild-type, Δ*nrdJ*, Δ*nrdD*, Δ*nrdJ*Δ*nrdD*, and Δ*cobN* strains was grown for 5 days in MM ± vitamin B_12_ (1 μg/ml). Biomass and thickness were calculated using COMSTAT software. Data are the average of three independent experiments. Asterisks over bars (^*^) indicate statistical differences (*p* < 0.005 in pairwise *t*-test calculated with GraphPad 6.0). **(B)** Pictures of the sum and the orthogonal views of biofilms stained with the LIVE/DEAD BacLight Bacterial Viability Kit before visualization by a confocal microscope. Images represent three independent experiments. Bars represent 200 μm.

Class II and III RNR enzymes are necessary for biofilm formation when class II RNR is highly expressed (Crespo et al., 2016). Currently, it is unknown whether vitamin B_12_ is synthesized and influences class II RNR activity under biofilm conditions. Thus, we analyzed different *P. aeruginosa* strains (wild-type and isogenic mutant strains for *nrdJ, nrdD* and *cobN* genes) grown in a continuous-flow cell biofilm system. Figure 5A shows that aerobic biofilm formation in minimal media, measured as total biofilm biomass and average thickness, decreased when class II and III RNR were mutated. We previously reported a similar result for biofilm cells grown in LB-rich media (Crespo et al., 2016). The *P. aeruginosa* Δ*cobN* mutant strain (vitamin B_12_ deficient) decreased in biofilm formation compared to the *P. aeruginosa* PAO1 strain, similar to the produced levels in any *P. aeruginosa* deficient for class II RNR (Δ*nrdJ* and Δ*nrdJ*Δ*nrdD*) (Figure 5A). Furthermore, the biomass and thickness levels in the *P. aeruginosa* PAO1 Δ*nrdJ* and Δ*nrdJ*Δ*nrdD* mutant strains did not reach the wild-type strain levels even when vitamin B_12_ was added_._ However, the *P. aeruginosa* PAO1 Δ*nrdD* and Δ*cobN* mutant strain biofilm thickness increased considerably when vitamin B_12_ was added, indicating active class II RNR activity.

These results suggest an active vitamin B_12_ biosynthesis in the *P. aeruginosa* biofilm growth via the *cobN* gene. Moreover, supplying vitamin B_12_ enabled optimum *P. aeruginosa* PAO1 biofilm growth in the biofilm layers without active vitamin B_12_ biosynthesis due to oxygen concentration strengths, activating class II RNR. As expected, cell filamentation morphology, in the Δ*nrdJ* mutants, was restored by adding vitamin B_12_ to the continuous-flow biofilm (Figure 5B).

### Vitamin B_12_ availability during *P. aeruginosa* aerobic and biofilm growth

We described that *P. aeruginosa* needs vitamin B_12_ availability during planktonic and biofilm growth, essential for class II RNR enzymatic activity. Thus, we elucidated for the first time the amount of vitamin B_12_ available for *P. aeruginosa* growth under different conditions (planktonic aerobic or anaerobic and biofilm) in different *P. aeruginosa* strains.

Quantifying vitamin B_12_ by HPLC-MS showed this molecule only in cells that were grown aerobically and in the stationary phase (Table 3). However, at exponential growth and in anaerobic conditions, vitamin B_12_ was detected below the technique detection limit, corroborating previous results under these conditions. Surprisingly, under 5-day-old continuous-flow biofilm *P. aeruginosa* PAO1 growth, cells produced a 10-fold increase in vitamin B_12_ compared to aerobic growth, indicating this biosynthetic pathway is activated under this circumstance. We suggested that vitamin B_12_ biosynthesis in biofilm is only produced in the upper-aerobic biofilm layer because we detected no vitamin B_12_ levels in cells grown anaerobically (Table 3). Some studies suggested that vitamin B_12_ (*cob*) aerobic synthesis genes are expressed more during biofilm growth (Anderson et al., 2008) in the mucoid phenotype (Rao et al., 2008) and the stationary phase (Fung et al., 2010), with downregulated anoxic conditions (Alvarez-Ortega and Harwood, 2007).

**Table 3:** Quantification of vitamin B_12_ levels by HPLC-MS.

	**ng Vitamin B_12_/mg protein**			
	**Aerobic exponential**	**Aerobic stationary**	**Anaerobic**	**Biofilm**
PAO1	0^nd^	0.32 ± 0.05	0^nd^	3.72 ± 0.01
PAET1	0^nd^	0.67 ± 0.06	0^nd^	1.72 ± 0.05
PA14	0^nd^	0.51 ± 0.02	0^nd^	0.84 ± 0.01

*Growth of P. aeruginosa PAO1, PAET1 and PA14 strains under aerobic (exponential OD550~0.5 and stationary OD550>2) and anaerobic conditions for 20 h (OD550~2) and under continuous-flow biofilm formation conditions (5 days). Values were normalized by protein concentration. Two independent experiments were performed, and the mean ± standard deviation is shown. nd, denotes Not-detected, below the technique detection limit*.

Previous studies showed different RNR activity in other *P. aeruginosa* strains under aerobic and anaerobic conditions (Crespo et al., 2017). Therefore, we analyzed vitamin B_12_ levels in strains more recently isolated, such as the *P. aeruginosa* PA14 and PAET1 strains, and we observed different vitamin B_12_ levels between strains. In *P. aeruginosa* PA14 and PAET1 strains, we identified increased vitamin B_12_ levels under aerobic conditions (1.6 and 2.1 times, respectively) (Table 3) and lower vitamin B_12_ levels under biofilm growth conditions compared to the *P. aeruginosa* PAO1 strain. These different vitamin B_12_ levels may affect RNR activity and expression (Figure 2), but further experiments are required to validate this hypothesis. It may be due to an active class III RNR detected in the most recently isolated strains compared to the *P. aeruginosa* PAO1 strain (Crespo et al., 2017).

### *P. aeruginosa* clinical isolates grow with hydroxyurea treatment

**Figure 6:**
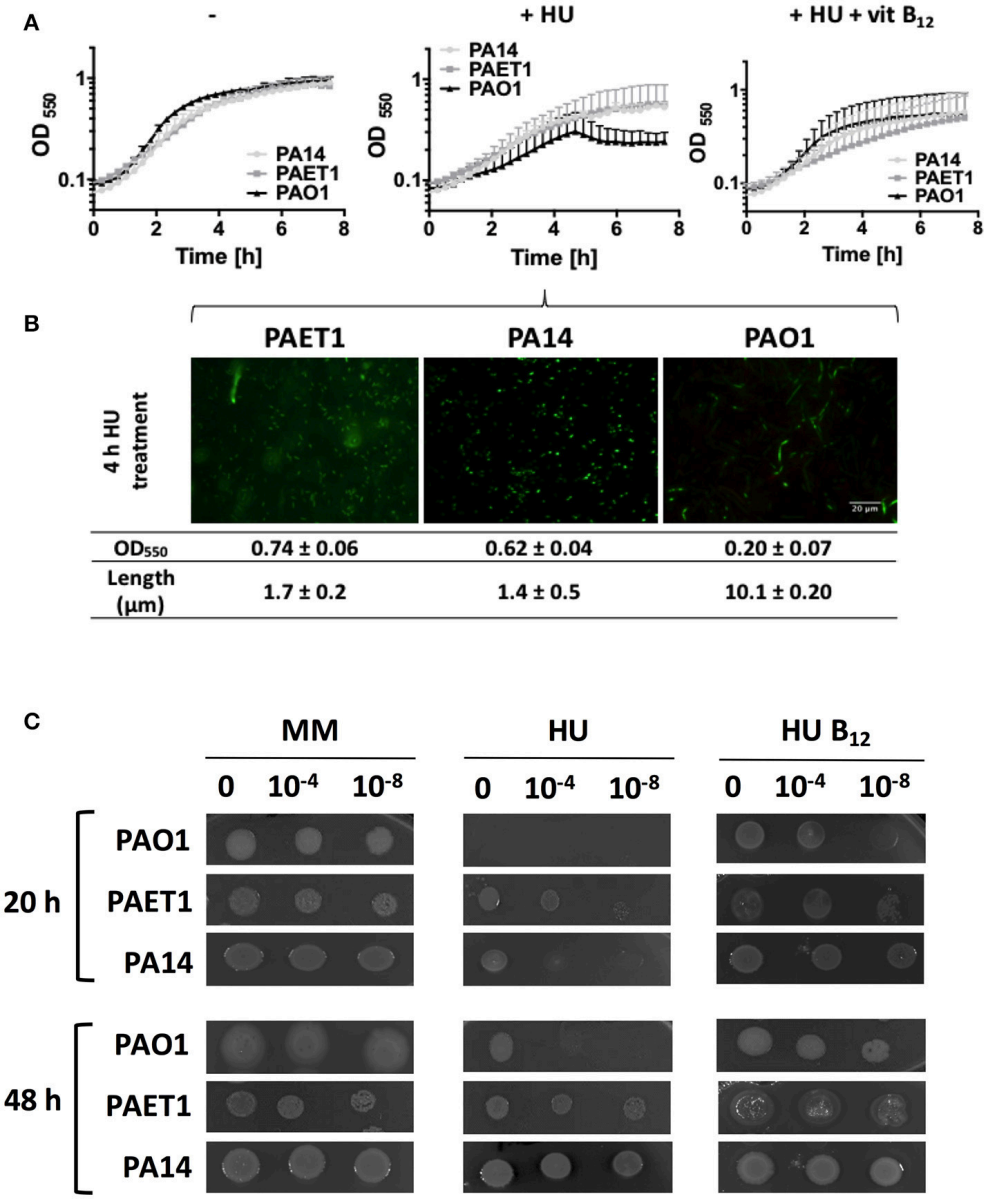
*P. aeruginosa* PAET1 and PA14 strains are resistant to HU. **(A)** Growth curve of *P. aeruginosa* PAO1, PAET1 and PA14 strains in MM containing 30 mM hydroxyurea (HU) and 1 μg/ml vitamin B_12_. **(B)** Cells were visualized 4 h after HU treatment. Images and values are representative of three independent experiments ± standard deviation. Bars represent 20 μm. **(C)** Aerobic *P. aeruginosa* PAO1, PAET1 and PA14 strain cell viability with HU and vitamin B_12_ at 20 or 48 h of incubation. Pictures represent three independent experiments. Cultures were diluted to 0, −4, and −8 for the growth.

Increased vitamin B_12_ availability in the aerobic growth of *P. aeruginosa* PA14 and the clinical isolate PAET1 strains suggests higher class II RNR activity under this growing condition. We evaluated strain growth in cells with class Ia RNR inhibited by adding 30 mM HU, which were only growing with an active class II RNR. The results showed that any *P. aeruginosa* strain could grow when vitamin B_12_ was added (Figure 6A). In contrast to *P. aeruginosa* PAO1, the absence of external vitamin B_12_ in *P. aeruginosa* PA14 and the clinical isolate PAET1 strain did not affect their growth aerobically. Therefore, vitamin B_12_ is more available in these strains than in the *P. aeruginosa* PAO1 strain for class II RNR activity.

Additionally, the cell morphology shown in the *P. aeruginosa* PAO1 strain after 4 h of HU treatment was filamentous (~10 μm). However, HU treatment of *P. aeruginosa* PA14 and PAET1 yielded rod-shaped cell morphology (1.4 and 1.7 μm), suggesting that their DNA replication was unimpaired (Figure 6B) after 4 h of treatment. However, *nrd* gene expression in *P. aeruginosa* clinical isolates strains was increased, suggesting that HU inhibited class I RNR as in the *P. aeruginosa* PAO1 strain (Table 4). This result was corroborated by analyzing their cell viability in a solid medium under HU treatment with and without vitamin B_12_. *P. aeruginosa* PA14 and PAET1 strains grew in as little as 20 h in the presence of HU (Figure 6C) compared with 48 h for *P. aeruginosa* PAO1.

**Table 4:** Expression of *nrdA, nrdJ*, and *nrdD* genes of *P. aeruginosa* PAO1 and clinical isolates (PAET1 and PA14) strains with HU.

	**Log-fold change (HU vs. H_2_O)**		
	**Class Ia *nrdA***	**Class Ia *nrdJ***	**Class III *nrdD***
PAO1	9.19 ± 2.1	31.65 ± 3	50.46 ± 7.2
PAET1	27.74 ± 2.1	7.63 ± 1.1	16.38 ± 0.5
PA14	4.91 ± 0.8	1.18 ± 0.2	32.7 ± 0.9

*Fold-change of nrd genes using qRT-PCR after 20 min of treatment with 30 mM HU compared with H_2_O. The gap gene was used as an internal standard. Two independent experiments were analyzed, and the standard deviation was annotated*.

## Conclusions

**Figure 7:**
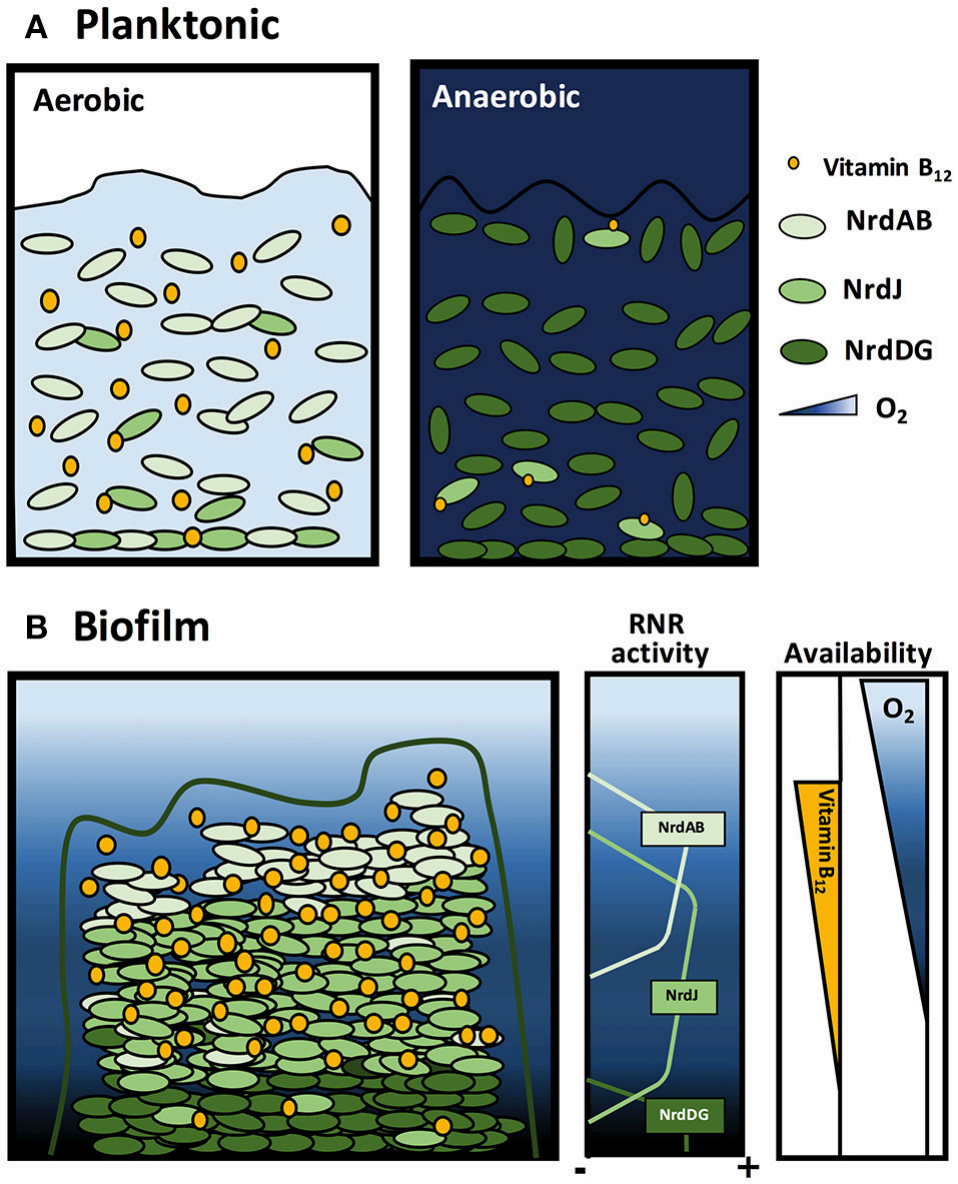
Model of ribonucleotide reductase activity and vitamin B_12_ availability during *P. aeruginosa* planktonic and biofilm growth. Scheme of *P. aeruginosa* growing in **(A)** aerobic and anaerobic planktonic cultures and **(B)** biofilm. Orange circles represent the vitamin B_12_ gradient, blue indicates oxygen concentration gradients and green indicates class Ia, II and III RNR activity.

We demonstrated that vitamin B_12_ synthesis occurs under *P. aeruginosa* aerobic planktonic growth conditions with an active class Ia RNR that supplies dNTPs required for DNA replication (Figure 7A). Vitamin B_12_ cannot be synthesized anaerobically when *P. aeruginosa* cells are grown with class III RNR (Figure 7A; Crespo et al., 2017). Class II RNR is enzymatically active when vitamin B_12_ is available through internal biosynthesis or from the environment.

*Pseudomonas aeruginosa* growing in biofilm differs and requires a more in-depth analysis. Oxygen diffusion through the complex biofilm structure generates an oxygen concentration gradient with apparent cell distribution with different RNR class activity (Figure 7B; Crespo et al., 2016). We suggest that in the superficial biofilm layers, aerobic cells express class Ia RNR, whereas in the internal layers, anaerobic conditions require cells to express class II or III RNR (Crespo et al., 2016). However, class II RNR is highly expressed during biofilm formation and in aerobic environments (Sjöberg and Torrents, 2011; Crespo et al., 2016) but is oxygen-independent and vitamin B_12_-dependent (aerobically synthetized). This leads us to ask under which conditions this RNR class enzymatically is active.

We suggest that external cells in a biofilm, which are in contact with aerobic environments, can synthesize vitamin B_12_, and it can diffuse through the biofilm structure creating a vitamin B_12_ concentration gradient along this structure. In this sense, class II RNR can be active in areas with microaerophilic conditions where class Ia or class III RNR are inactive (Figure 7B). Consequently, these results bring us closer to understanding the *P. aeruginosa* cell division mechanism through dNTP synthesis in planktonic and biofilm conditions.

## Author contributions

AC, NB-C, and ET: designed the study; AC and NB-C: performed the experiments. All authors analyzed the data, wrote the paper, and read and approved the final version.

### Conflict of interest statement

The authors declare that the research was conducted in the absence of any commercial or financial relationships that could be construed as a potential conflict of interest.
